# Diabetes distress among adults with type 2 diabetes mellitus in the North of West Bank-Palestine

**DOI:** 10.1007/s44192-025-00304-4

**Published:** 2025-11-25

**Authors:** Ibrahim Ghoul, Mohammed F. Hayek, Yousef Ahwal, Shahd Abdalghani, Yazeed Abo Baker, Malak Abed Aljwad, Eman Alshawish, Mohamed Kbalan, Abdullah Abdullah, Nizar B. Said, Aidah Alkaissi, Murad Jkhlab

**Affiliations:** 1 Oncology and Hematology department, An-Najah National University Hospital, An-Najah National University, Nablus, Palestine; 2 Faculty of Medicine and Health Sciences, Nursing and Midwifery Department, An-Najah National University, Nablus, Palestine; 3 Quality and Patient Safety Department, An-Najah National University Hospital, An-Najah National University, Nablus, Palestine; 4 Urology Center of Excellence, An-Najah National University Hospital, An-Najah National University, Nablus, Palestine

**Keywords:** Diabetes distress, Type 2 diabetes mellitus, North of West Bank Palestine, Psychological burden, Glycemic control, Socioeconomic factors, Cross-sectional study

## Abstract

**Background:**

Diabetes distress, the emotional burden and stress related to managing type 2 diabetes mellitus, has been linked to poor self-management and adverse health outcomes. In Palestine, particularly in the North of West Bank, adults with type 2 diabetes mellitus face unique social and economic challenges that may exacerbate diabetes distress, impacting their ability to maintain effective diabetes management. Addressing diabetes distress is crucial for improving health outcomes and quality of life in this population, yet research on its prevalence and associated factors in the North of West Bank is limited. This study aimed to assess the prevalence of diabetes distress and identify its associated demographic, socioeconomic, and clinical factors among adults with type 2 diabetes mellitus in the North of West Bank, Palestine.

**Methodology:**

A cross-sectional study was conducted with 404 adults diagnosed with type 2 diabetes mellitus in various healthcare centers across the North of West Bank. Participants completed the Diabetes Distress Scale-17 and a sociodemographic questionnaire, and additional clinical data such as HbA1c levels. Descriptive and inferential statistics were employed to identify associations between diabetes distress and demographic, socioeconomic, and clinical factors, with a focus on assessing the impact of these variables on distress levels.

**Results:**

Of the 423 eligible individuals approached, 404 participants were included in the final analysis (response rate: 95.5%). The median age was 55 years (IQR: 49–63), with a nearly equal gender distribution. Poor glycemic control (HbA1c > 6.4%) was observed in 76.0% of participants, and 74.3% reported one or more comorbidities. High and moderate levels of diabetic distress were reported by 29.0% and 25.2% of participants, respectively, with emotional and regimen-related distress being the most prevalent domains. Significant factors associated with higher distress included comorbidities, smoking, urban residence, unemployment, living with family, and residence in Nablus. Multinomial logistic regression revealed that absence of comorbidities, younger age, living alone, and non-urban residence were protective factors against high distress (*p* < .05). The model explained 20.1% of the variance in distress levels (Nagelkerke R² = 0.201).

**Conclusion:**

Diabetes distress is common among adults with type 2 diabetes and is significantly influenced by clinical, sociodemographic, and lifestyle factors. Routine screening and targeted psychosocial interventions are essential, especially for high-risk groups, to improve both psychological well-being and diabetes outcomes.

## Background

Type 2 Diabetes Mellitus (T2DM) is a chronic metabolic disorder that requires lifelong self-management, including adherence to dietary plans, physical activity, medication regimens, and frequent blood glucose monitoring [1]. These sustained demands can lead to a psychological condition known as diabetes distress characterized by frustration, worry, and burnout due to the burden of managing the disease [2]. Diabetes distress is distinct from clinical depression and has been shown to adversely affect treatment adherence and glycemic control [3], ultimately impacting patients’ quality of life [4]. Globally, diabetes distress is recognized as a key factor affecting diabetes management [5–7]. However, its prevalence and associated risk factors vary significantly across populations, with reported rates ranging from 17% to over 60%. For example, a 2021 study conducted among Indian patients with type 2 diabetes mellitus reported a 42% prevalence of diabetes distress, with regimen-related distress being the most common subtype [8]. Similarly, another cross-sectional study from Saudi Arabia in 2021 found a prevalence of 48.5% among adult patients with type 2 diabetes, also exploring psychosocial predictors of distress in this population [9], while a global systematic review estimated it at around 36% [10, 11]. Several clinical and psychosocial factors have been associated with higher levels of diabetes distress, including insulin use [12], recent hypoglycemic episodes [13], diabetes complications such as retinopathy [13], and limited family or social support [12]. Demographic variables such as lower education, unemployment, and inadequate income also contribute to elevated distress [13].

In regions with limited healthcare infrastructure, such as the North of West Bank in Palestine, the emotional burden of managing diabetes may be intensified by economic hardship, unstable political conditions, and cultural expectations. Despite this context, there is a lack of research focusing on diabetes distress among Palestinian adults. Few studies have explored how socioeconomic and geographic factors influence diabetes distress in Arab or Palestinian populations. Some studies have examined the psychological burden in patients with both type 1 and type 2 diabetes, noting that distress levels can be comparable across both groups [14]. However, this study focuses exclusively on adults with T2DM, as it remains the most prevalent type of diabetes in Palestine and presents distinct self-management challenges in older adult populations compared to youth or young adults with type 1 diabetes.

This study addresses a critical gap in the literature by investigating the prevalence of diabetes distress and identifying its associated demographic, socioeconomic, and clinical factors among adults with type 2 diabetes in the North of West Bank, Palestine. Findings from this study aim to guide healthcare providers and policymakers in developing culturally responsive interventions to reduce diabetes distress and enhance diabetes management in resource-limited settings.

## Methodology

### Study design and settings

A cross-sectional study conducted from November 2024 to February 2025 across selected healthcare centers in the North of West Bank, Palestine, reflects the region’s limited healthcare infrastructure, fragmented referral pathways, economic constraints, and persistent socio-political instability, all of which place additional burdens on chronic disease management. Several studies have highlighted how systemic issues such as limited access to recommended medications, inconsistent application of clinical guidelines, and fragmented care delivery have negatively impacted diabetes management and contributed to poor health outcomes in Palestinian healthcare settings [15–18]. Furthermore, cultural expectations and familial obligations significantly influence patient behaviors and access to care, contributing to unique psychosocial stressors for individuals with diabetes [19–21]. These factors collectively impact diabetes management and underscore the need for tailored interventions that address both structural and sociocultural barriers in this context [15, 18, 19, 21].

### Sample and sampling

The estimated population of adults with T2DM in Palestine was 388,420, based on the Health Annual Report Palestine 2023 (Ministry of Health, 2023). The required sample size was calculated using the Raosoft online calculator, assuming a 95% confidence level, 5% margin of error, and a response distribution of 50% to account for maximum variability due to the lack of local prevalence estimates. Although existing literature reports DD prevalence between 17% and 60%, the 50% estimate was used for conservative sample estimation. The calculated sample size was 384 participants; to ensure adequate power and account for non-responses, the final sample included 404 participants. This was a facility-based study conducted in government primary healthcare clinics and specialized diabetes centers in five major districts: Nablus, Tulkarm, Jenin, Qalqilya, and Tubas. Participants were selected using a convenience sampling method. The majority of the sample was drawn from patients attending a major healthcare facility in Nablus, where data collection was primarily conducted. Additional participants were included from surrounding governorates such as Tulkarm, Qalqilya, Jenin, and Tubas, based on accessibility and availability during the data collection period. This approach resulted in a sample distribution that was heavily concentrated in Nablus, with smaller proportions from other cities. While this method facilitated efficient data collection, it may limit the generalizability of the findings to the broader population of patients with type 2 diabetes across the West Bank.

### Eligibility criteria

Participants were eligible if they were aged 18 years or older, had a documented diagnosis of T2DM for at least six months, and were cognitively and mentally able to provide informed consent. Individuals with severe mental illness or cognitive impairment were excluded. Only T2DM patients were included; the differentiation from T1DM was made based on physician diagnosis documented in the medical record and clinical characteristics such as age of onset, insulin initiation timeline, and body mass index. Patients with unclear classification or inconsistent records were excluded to minimize misclassification bias.

### Data collection and study tools

Data were collected using a self-administered, paper-based questionnaire administered during clinic visits. The questionnaire consisted of three parts:

- Sociodemographic information (age, gender, education, marital status, employment, residence).
- Clinical history (treatment type, comorbidities, family history, smoking status, recent hyperglycemia or hypoglycemia).
- The Arabic version of the Diabetes Distress Scale-17 (DDS-17), which includes four domains: emotional burden (5 items), physician-related distress (4 items), regimen-related distress (5 items), and interpersonal distress (3 items). Each item is rated on a 6-point Likert scale from 1 (no distress) to 6 (severe distress). The overall mean score was interpreted as follows: <2.0 = little or no distress; 2.0–2.9 = moderate distress; ≥3.0 = high distress [22].

The Arabic version of the DDS-17 was used [23]; The Cronbach’s alpha value was 0.848 for the total scale. The test-retest reliability value was 0.78 [9]. Clinical data, including HbA1c values, treatment type, and comorbidities, were extracted from patient medical records by trained clinic staff (nurses or physicians) with the patient’s consent. Only HbA1c results from the past 3 months were accepted; values older than 90 days were excluded from the analysis to maintain current glycemic relevance. Sociodemographic and clinical data were obtained through patient interviews and verified from medical records (for comorbidities, HbA1c, and treatment type).

### Operational definitions

Diabetes duration was categorized as ≤ 5 years or >5 years based on patient records; comorbidities were defined as the presence of one or more additional chronic conditions documented in the medical record; smoking status was classified as current smoker (daily or occasional use) or non-smoker; residence was categorized into city, village, or refugee camp according to official address; occupation was grouped into employed, unemployed, retired, or student; and levels of diabetic distress were determined using the DDS-17 scale with cutoffs of < 2 indicating little or no distress, 2.0–2.9 indicating moderate distress, and ≥ 3.0 indicating high distress. Routine follow-up visits are defined as attending the health facility at least once every three months, in line with national diabetes care guidelines. Glycemic control is assessed by measuring HbA1c levels and categorized as follows: HbA1c < 5.7% is considered normal, 5.7–6.4% indicates prediabetes, and ≥ 6.5% reflects poor glycemic control, consistent with ADA and WHO guidelines. Regular follow-up and adherence to clinic visits are strongly associated with improved glycemic control, while missed appointments and poor adherence significantly increase the risk of poor glycemic outcomes in patients with diabetes [24–27].

### Data entry and analysis

All responses were manually entered into Microsoft Excel and then imported into SPSS version 21 (IBM Corp., Armonk, NY, USA) for statistical analysis. Double-entry verification was performed to minimize entry errors. Descriptive statistics were used to summarize the sample characteristics. Associations between categorical variables and diabetes distress were analyzed using chi-square tests, with significance set at *p* < .05, with standardized residuals (± 1.96) used post hoc to identify key subgroup differences; some variables were regrouped into two categories for analysis. Multinomial logistic regression was performed using high distress as the reference category to estimate adjusted odds ratios.

### Ethical considerations

This study was approved by the Institutional Review Board (IRB) at An-Najah National University and was conducted in accordance with the ethical principles of the Declaration of Helsinki. Informed written consent was obtained from all participants after explaining the purpose, procedures, voluntary nature, and confidentiality of the study. Personal identifiers were removed to ensure anonymity. Permission was obtained from the Ministry of Health and directors of participating clinics to conduct the research.

## Results

### Participant inclusion and response rate

A total of 423 eligible individuals were approached across selected health centers. After excluding 13 who declined participation and 6 with incomplete responses, 404 participants were included in the final analysis, yielding a response rate of 95.5%.

### Sociodemographic and personal characteristics of study participants

The study included 404 adults with nearly equal gender distribution (50.7% males and 49.3% females) and a median age of 55 years (IQR: 49–63), with the largest age group being 40–60 years (53.5%). Regarding education, 38.6% had completed secondary school, and in terms of employment, 47.5% were unemployed while 40.3% were employed. The majority were married (75.2%) and lived with family members (90.8%). Geographically, participants were primarily from urban (47.0%) and rural (45.3%) areas, with 77.0% residing in Nablus (Table 1).

**Table 1:** Sociodemographic and personal characteristics of study participants (*N* = 404)

		Percent	Count
Sex	Female	49.3%	199
	Male	50.7%	205
Age	18–39	12.6%	51
	40–60	53.5%	216
	> 60	33.9%	137
Level of education	Uneducated	9.4%	38
	Primary	21.3%	86
	Secondary	38.6%	156
	Diploma	10.6%	43
	Bachelor	15.8%	64
	Postgraduate	4.2%	17
Occupation	Student	4.2%	17
	Unemployed	47.5%	192
	Retired	7.9%	32
	Employed	40.3%	163
Marital status	Widowed	11.9%	48
	Single	9.7%	39
	Married	75.2%	304
	Divorced	3.2%	13
Live with family	With family	90.8%	367
	Alone	9.2%	37
Residence	Village	45.3%	183
	Refugee Camp	7.7%	31
	City	47.0%	190
City	Jenin	4.7%	19
	Tubas	1.7%	7
	Tulkarm	9.4%	38
	Qalqilya	7.2%	29
	Nablus	77.0%	311

### Clinical-related characteristics of study participants

The clinical characteristics of the participants (Table 2) indicate that the majority (71.8%, *n* = 290) had been living with type 2 diabetes for more than five years with a median duration of 7 years (IQR: 5–16), and 74.3% (*n* = 300) had one or more comorbidities. Regarding treatment regimens, 44.3% (*n* = 179) used non-insulin therapies, while 35.4% (*n* = 143) were on insulin and 20.3% (*n* = 82) on a combination. Routine health center visits were reported by 64.6% (*n* = 261), and 58.2% (*n* = 235) experienced hyperglycemia in the past month, while 54.0% (*n* = 218) reported hypoglycemic events. Most participants (76.0%, *n* = 307) had HbA1c levels above 6.4, suggesting poor glycemic control. Additionally, 83.2% (*n* = 336) had a family history of diabetes, 42.6% (*n* = 172) were smokers, and 57.4% (*n* = 232) were non-smokers. 58.2% and 54.0% reported hyperglycemia and hypoglycemia events, respectively, in the previous month.

**Table 2:** Clinical-related characteristics of study participants (*N* = 404)

		Percent	Count
Duration with diabetes2	=<5	28.2%	114
	> 5 Years	71.8%	290
Treatment regiment	Insulin	35.4%	143
	Non-insulin	44.3%	179
	Combination	20.3%	82
Visit health center for routine follow up	No	35.4%	143
	Yes	64.6%	261
HbA1c	< 5.7	12.6%	51
	5.7–6.4	11.4%	46
	> 6.4	76.0%	307
Hyperglycemia event in last 1 month	No	41.8%	169
	Yes	58.2%	235
Hypoglycemia event in last 1 month	No	46.0%	186
	Yes	54.0%	218
Family history	No	16.8%	68
	Yes	83.2%	336
Smoking Status	No	57.4%	232
	Yes	42.6%	172
Comorbidities	No	25.7%	104
	Yes	74.3%	300

### Prevalence of diabetic distress

The overall prevalence of diabetes distress was considerable. Nearly one-third of participants (29.0%) experienced high levels of distress, and an additional 25.2% reported moderate distress. In contrast, 45.8% had no or little distress. Across the four distress domains, emotional distress was the most common, with 34.9% of participants experiencing it at a high level and 40.1% at a moderate level. Regimen-related distress followed, reported as high by 30.9% and moderate by 32.2% of participants. Physician-related distress was reported as high by 28.0% and moderate by 15.6%, while interpersonal distress was less prevalent, with 23.8% reporting high levels and 16.8% reporting moderate levels (Table 3).

**Table 3:** Prevalence of Diabetic Distress (*N* = 404)

		Percent	Count	
Emotional distress	No-Little	25.0%	101	
	Moderate	40.1%	162	
	High	34.9%	141	
Physician distress	No-Little	56.4%	228	
	Moderate	15.6%	63	
	High	28.0%	113	
Regimen distress	No-Little	36.9%	149	
	Moderate	32.2%	130	
	High	30.9%	125	
Interpersonal distress	No-Little	59.4%	240	
	Moderate	16.8%	68	
	High	23.8%	96	
Diabetic distress	No-Little	45.8%	185	
	Moderate	25.2%	102	
	High	29.0%	117	

### Factors associated with diabetic distress among patients with type 2 diabetes

The analysis of factors associated with diabetic distress revealed several significant associations with sociodemographic, clinical, and lifestyle variables (Table 4). Occupation was significantly associated with distress (*p* < .001), as were marital status (*p* = .002), living arrangements (*p* = .024), and residence (*p* < .001). The presence of comorbidities (*p* = .035) and smoking status (*p* = .027) were also significantly linked to distress. Other factors, including gender, age, education level, diabetes duration, treatment regimen, routine health visits, HbA1c levels, and family history, did not show significant associations (*p* > .05). These results indicate that certain sociodemographic and lifestyle variables were statistically associated with distress, although chi-square tests do not specify the direction or magnitude of differences across subgroups (Table 4).

**Table 4:** Factors associated with Diabetic distress among patients with type 2 diabetes (N = 404)

Variable	*N*	No-Little Distress %	Moderate Distress %	High Distress %	*P*-value
Sex	404				0.134
Female	199	47.2%	28.1%	24.6%	
Male	205	44.1%	22.4%	33.5%	
Age Category	404				0.097
18–39	51	39.2%	15.7%	45.1%	
40–60	216	49.1%	24.1%	26.9%	
> 60	137	41.6%	29.2%	29.2%	
Education Level	404				0.211
Uneducated	38	47.4%	21.1%	31.6%	
Primary	86	34.9%	29.1%	36.0%	
Secondary	156	51.3%	26.3%	22.4%	
Diploma	43	48.8%	23.3%	27.9%	
Bachelor	64	42.2%	23.4%	34.4%	
Postgraduate	17	58.8%	11.8%	29.4%	
Occupation	404				0.000*
Student	17	0.0%	5.9%	94.1%	
Unemployed	192	45.3%	28.1%	26.6%	
Employed	163	53.4%	20.7%	25.8%	
Retired	32	40.6%	28.1%	31.3%	
Marital Status	404				0.002*
Single	39	33.3%	12.8%	53.8%	
Married	304	49.0%	26.0%	25.0%	
Widowed	48	43.8%	31.3%	25.0%	
Divorced	13	46.2%	15.4%	38.5%	
Living Status	404				0.024*
With Family	367	46.3%	26.4%	27.2%	
Alone	37	43.2%	10.8%	45.9%	
Residence	404				0.000*
City	190	54.7%	20.0%	25.3%	
Village	183	38.3%	32.2%	29.5%	
Refugee Camp	31	45.2%	12.9%	41.9%	
City	404				0.000*
Nablus	311	53.1%	24.1%	22.8%	
Tulkarm	38	44.7%	31.6%	23.7%	
Qalqilya	29	41.4%	24.1%	34.5%	
Jenin	19	36.8%	26.3%	36.8%	
Tubas	7	57.1%	14.3%	28.6%	
Duration with Diabetes	404				0.265
≤ 5 years	158	39.9%	18.4%	41.8%	
> 5 years	246	47.2%	26.8%	26.0%	
Treatment Regimen	404				0.097
Non-insulin	226	46.9%	22.1%	31.0%	
Insulin	148	45.3%	22.3%	32.4%	
Combination	30	40.0%	23.3%	36.7%	
Routine Follow-up	404				0.701
Yes	332	47.3%	24.4%	28.3%	
No	72	41.7%	22.2%	36.1%	
HbA1c Level	404				0.365
< 5.7	33	60.6%	18.2%	21.2%	
5.7–6.4	45	37.8%	13.3%	48.9%	
> 6.4	326	44.2%	25.2%	30.7%	
Hyperglycemia in Last Month	404				0.094
Yes	233	42.1%	24.0%	33.9%	
No	171	48.5%	25.7%	25.7%	
Hypoglycemia in Last Month	404				0.966
Yes	190	42.6%	25.3%	32.1%	
No	214	47.2%	23.8%	29.0%	
Family History of Diabetes	404				0.160
Yes	340	46.2%	23.5%	30.3%	
No	64	43.8%	20.3%	35.9%	
Smoking Status	404				0.027*
Yes	161	38.5%	16.1%	45.3%	
No	243	50.6%	25.1%	24.3%	
Comorbidities	404				0.035*
Yes	277	42.6%	24.2%	33.2%	
No	127	51.2%	24.4%	24.4%	

Frequencies may not perfectly sum to the totals reported in Table 3 due to rounding of percentages.

### Multinomial logistic regression and effect size analysis of factors associated with diabetic distress

Multinomial logistic regression was conducted to explore the predictors and effect sizes of factors associated with diabetic distress, using high distress as the reference category. The overall model was statistically significant (χ² = 78.586, df = 42, *p* = .001), with a Nagelkerke R² of 0.201, indicating that approximately 20.1% of the variance in distress levels was explained by the included variables. The analysis revealed several significant predictors with notable effect sizes. Participants without comorbidities were significantly more likely to report lower distress levels compared to those with comorbidities, with an odds ratio (OR) of 3.13 for moderate distress (*p* = .005) and 2.47 for no/little distress (*p* = .010), indicating strong effect sizes. Those aged 18–39 had lower odds of reporting moderate distress compared to the 40–60 age group (OR = 0.35, *p* = .048), suggesting a higher likelihood of severe distress among younger individuals, rather than a protective effect of younger age. Living alone was associated with lower odds of reporting moderate distress compared to living with family (OR = 0.24, *p* = .020), while living outside the city (in villages or refugee camps) was associated with significantly higher odds of experiencing high distress (OR = 0.48, *p* = .006). These odds ratios serve as direct indicators of effect size and demonstrate how sociodemographic and clinical factors influence the probability of distress among individuals with type 2 diabetes (Table 5).

**Table 5:** Multinomial Logistic Regression and Effect Size Analysis (N = 404)

Predictor	Comparison Group	Category Compared To	B (Coef.)	Sig. (*p*)	OR (Exp(B))	95% CI (OR)	Interpretation
Comorbidities	Moderate	Yes	1.141	0.005	3.13	1.42–6.90	No comorbidity → 3x higher odds of moderate vs. high distress
Comorbidities	No/Little	Yes	0.906	0.010*	2.47	1.24–4.94	No comorbidity → 2.5x higher odds of no/little vs. high distress
Age (18–39 vs. 40–60)	Moderate	40–60	− 1.048	0.048	0.35	0.12–0.99	Younger age (18–39) less likely to report moderate distress
Living with family	Moderate	Yes	− 1.415	0.020*	0.243	0.074–0.802	Living alone → lower odds of moderate vs. high distress
Living in city	No/Little	City	− 0.733	0.006*	0.48	0.28–0.81	Living in village/camp → lower odds of high distress

*Significant Predictors of Diabetic Distress (Reference = High Distress)

### Post hoc analysis using standardized residuals: identifying subgroups associated with diabetic distress

The chi-square analysis revealed significant associations between diabetic distress levels and several variables, including comorbidities (*p* = .035), smoking status (*p* = .027), occupation (*p* = .03), living with family (*p* = .024), and living in the city (*p* = .003). Post hoc examination of standardized residuals indicated that participants with comorbidities and smokers were more likely to experience high distress than expected. Those living in cities showed significantly higher levels of moderate and high distress, while those in rural areas reported lower distress than expected. Individuals not living with their families and those unemployed or in informal occupations also tended to have higher distress levels. These post hoc findings suggest that certain subgroups may be particularly vulnerable to psychological burden related to diabetes, warranting targeted interventions. No significant associations were found with age, sex, education, HbA1c, glycemic events, diabetes duration, insulin use, or family history (Table 6).

**Table 6:** Summary of Chi-square Results with Post Hoc Interpretation (N = 404)

Variable	χ² (df)	*p*-value	Post Hoc Findings Based on Standardized Residuals ( ≥ ± 1.96)
Comorbidities	6.72 (2)	0.035	more high distress with comorbidities, but not significant (+ 1.1)
Smoking Status	7.20 (2)	0.027	High distress more frequent in smokers (+ 1.4), moderate distress less in smokers (− 1.4)
Occupation	6.48 (2)	0.039	No-little distress less frequent among unemployed (+ 1.4), more among employed (-1.2)
Live with Family	7.48 (2)	0.024	High distress more frequent among those not living with family (+ 1.9)
Live in City	11.87 (2)	0.003	No-little distress more frequent in urban (+ 1.4), moderate distress more frequent in rural (− 1.7)
Family History	3.66 (2)	0.160	Not significant
HbA1c	4.31 (4)	0.365	Not significant
Sex	4.03 (2)	0.134	Not significant
Level of Education	13.23 (10)	0.211	Not significant
(Other variables)	–	–	Not significant or no post hoc residuals ≥ ± 1.96

Residuals < ± 1.96; findings reflect trends, not statistically significant differences.

## Discussion

The present study provides a comprehensive examination of the prevalence and determinants of diabetic distress among adults with type 2 diabetes, revealing that nearly one-third of participants experienced high levels of distress, with emotional and regimen-related domains being most prominent. These findings are consistent with a growing body of international literature that underscores the high burden of diabetes distress in this population, with reported prevalence rates ranging from 17% to over 60% depending on the setting, measurement tools, and population characteristics [28–33]. The predominance of emotional and regimen-related distress aligns with previous studies, which have identified these domains as central to the lived experience of diabetes distress [11, 34, 35].

A notable contribution of this study is the identification of sociodemographic and lifestyle factors such as occupation, marital status, living arrangements, urban residence, comorbidities, smoking, and geographic location as significant predictors of distress. These results echo findings from diverse settings, where unemployment, being married, living with family, and rural residence have been linked to higher distress levels [11, 30, 36–40]. The association between comorbidities and increased distress is particularly robust, as supported by both the current analysis and prior research, which consistently highlight the compounding psychological burden of multiple health conditions [29, 36, 37, 39, 41, 42]. The significant relationship between smoking and distress also mirrors previous reports, suggesting that lifestyle risk factors may both contribute to and result from psychological distress in diabetes [37, 43, 44].

Interestingly, the study found that clinical variables such as glycemic control (HbA1c), diabetes duration, treatment regimen, and recent glycemic events were not significantly associated with distress in this cohort. This finding diverges from some studies that have reported strong links between poor glycemic control, insulin use, and higher distress [38, 41, 45–50]. However, other research has similarly failed to find consistent associations, suggesting that the relationship between clinical status and distress may be context-dependent or mediated by psychosocial factors [28, 51–53]. The lack of association with family history and education level further highlights the complex, multifactorial nature of diabetes distress, where social and psychological determinants may outweigh traditional clinical predictors in certain populations [32, 36, 38, 51, 54–57].

The multinomial logistic regression and post hoc analyses reinforce the importance of social context, revealing that living alone, and urban residence are protective against moderate and high distress, while comorbidities and rural living increase risk. Younger age (18–39) was associated with a higher likelihood of severe distress compared to middle-aged participants, which contrasts with some studies reporting protective effects of younger age, younger adults may experience greater psychological burden due to career, family, or social pressures. These nuanced findings are in line with recent network and cluster analyses, which emphasize the heterogeneity of distress profiles and the need for tailored interventions [11, 34, 58]. The identification of vulnerable subgroups such as smokers, those with comorbidities, and rural residents provides actionable targets for screening and intervention, as recommended by international guidelines and supported by meta-analytic evidence [33, 59, 60].

Despite the strengths of a high response rate and robust analytic approach, several limitations should be acknowledged. The cross-sectional design precludes causal inference, and the reliance on self-reported measures may introduce reporting bias. The study population, drawn primarily from a single geographic region, may limit generalizability to other settings with different cultural, healthcare, or socioeconomic contexts. Additionally, while the model explained a meaningful proportion of variance in distress, a substantial amount remains unexplained, pointing to the likely influence of unmeasured psychological, environmental, or systemic factors [28, 58, 61, 62].

Future research should prioritize longitudinal designs to clarify causal pathways and temporal relationships between distress and its predictors. There is also a need for qualitative and mixed-methods studies to deepen understanding of the lived experience of diabetes distress, particularly in underrepresented and high-risk subgroups. Intervention studies targeting modifiable risk factors such as comorbidity management, smoking cessation, and social support enhancement are warranted to evaluate their impact on distress and related health outcomes [29, 61, 63–65]. Finally, integrating routine distress screening into diabetes care, as advocated by recent guidelines, may facilitate early identification and holistic management of psychological burden in this population [30, 33, 56, 59].

In summary, this study adds to the growing evidence that diabetes distress is a prevalent and multifaceted challenge among adults with type 2 diabetes, shaped by a complex interplay of sociodemographic, clinical, and lifestyle factors. Addressing these determinants through targeted, context-sensitive interventions holds promise for improving both psychological well-being and diabetes outcomes.

## Conclusion

In conclusion, diabetes-related distress was found to be highly prevalent among adults with type 2 diabetes in this study population and appeared to be influenced by a range of sociodemographic and clinical factors. Notably, associations were observed with variables such as comorbidities, occupation, marital status, living arrangements, smoking, residence type, and younger age, underscoring the multifactorial nature of distress in this population. Emotional and regimen-related distress were particularly common. While causal relationships cannot be established, these findings highlight the value of routine screening for diabetes distress and suggest that incorporating psychosocial support into care may benefit patients in similar settings. Future research using longitudinal and interventional designs is recommended to clarify these associations and guide targeted interventions.

## Data Availability

The data that support the findings of this study are available from the corresponding author upon a reasonable request.

## Abbreviations

T2DM: Type 2 diabetes mellitus
